# Clinical features as discriminating factors in the choice of tracheostomy techniques

**DOI:** 10.1186/1471-2482-13-S1-A28

**Published:** 2013-09-16

**Authors:** Lucia Marullo, Giuseppe Izzo, Annarita Orsini, Jole Petruzzi, Anna d’Elia, Luana Vessicchio, Fausto Ferraro

**Affiliations:** 1 Dept. of Anesth. Surgical and Emergency Sciences-Intensive Care Unit II University of Naples, Italy

## Introduction

Tracheostomy is a generally accepted procedure that assures free access to the airways in long-term lung ventilation. Apart from surgical tracheostomy, percutaneous dilational tracheostomy (PDT) has been increasingly employed in intensive care units (ICU). There is no optimal tracheostomy (TS) technique and in comparisons of percutaneous techniques, no single technique has proved to be superior. For this reason, the operator's skill and experience and the clinical anatomical and physiopathological features of the patient should be discriminating factors in the choice of TS technique. Because of this we developed a decisional making algorithm as a guide to choose tracheostomy method more adapted to critical patients' characteristics. It’s use in elective TS in ICU seems to reduce complications in our exeprience, regarding leterature data.

## Background

TS is an elective technique used in ICU for the management of patients requiring prolonged mechanical ventilation (MV) [1]. When elective tracheotomy is indicated in critically ill patients, the technique of PDT offers important advantages over surgical tracheosthomy (ST). With PDT, less clinically significant wound infection is observed compared with ST, probably due to minimisation of local tissue trauma and a tighter fit between cannula and the surrounding tissues. Nowadays more PDT methods are in use: TLT, Ciaglia blue-Rhino, PercuTwist [1], Ciaglia Blue-Dolphin. But considering the clinical and anatomical variability of ICU patients, there is no ideal risk-free TS technique. Operator's experience and patient's physiopathological characteristics should be always considered [2]. Because of this our dedicated team have outlined a decisional algorithm to choose the most appropriate technique in each case (Table 1) to reduce the incidences of complication.

**Table 1:** Algorithm complications vs Literature complications.

Tracheostomy Technique	CBD	BC	CBR	P-Twist	TLT	Surgical	Total	Study group	Literature
**Patients n.**	**16**	**2**	**44**	**9**	**15**	**2**	**118**		

**Complications**								**%**	**%**

Bleeding	1	0	2	0	0	0		2.50%	1,2% - 4,65%
Ring fractures	3	0	0	8	0	0		10.20%	?
Stenosys	0	0	2	0	1	0		2.50%	0,8% - 3,7%
Edema	3	0	3	0	0	0		5.00%	3.40%
Tracheoesofagea fistula	1	0	0	0	0	0		0.85%	?
Pneumothorax	0	0	0	0	0	0		0.00%	0.4-3%
Subcutaneous emphysema	0	0	0	0	0	0		0.00%	1312.00%

## Method

**Figure 1:**
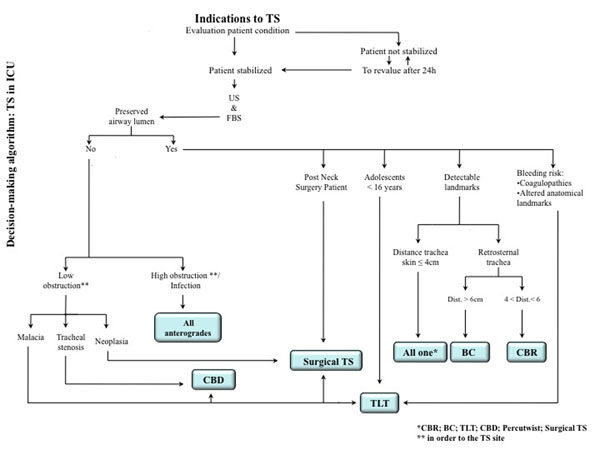


We have considered consecutive 118 patients, admitted in a general intensive care of our University Hospital in the last three years, skeduled for tracheostomy. Generally in our practice TS is performed by residents after a specific training program under the guidance and supervision of a senior tutor with specific skills. Each patient was evaluated by ultra sounds (US), and video-bronchoscopy (video-FBS). Algorithm was formulated by our ten-years-experince with TS techniques, comparing the specific characteristics of each technique to the physiopathological characteristics of each patient. Figure 1.

## Results and discussion

We recorded complications related to tracheostomy performed, applying the decisional making algorithm, in the last 3 years.

Comparing with the complications reported in the literature [3-5] (Table 1), the use of our decisional flow-chart as guide to choose the kind of PDT thecnique seems to reduce the incidence of complications considered (Table 1).

The systematic use of U.S. and video-FBS facilitates the execution of the TS and reduces complications [3]. In our observational retrospective study we have a small case series without control group; it is insufficient for a statistical validation of the findings (118 patients in the last three years), even if quantitatively similar with other monocentric study in literature. Furthermore, in our general university ICU polytrauma and cardiac surgical patients are less frequent then septic, surgical or COPD patients. Because of this our decisional making algorithm should be evaluated on a more heterogeneous population. The application of our algorithm required knowledge, skill and availability of at least three techniques: surgical, intrusive and extrusive percutaneous oncewith costs increased. But the reduction of complications would lead to a reduction in ICU stay and in health care costs [2].

## Conclusions

The application of our algorithm to choose which kind of TS technique was more adapt to the peculiarities of each patient, seems to reduce incidence of complications, compared to the incidence reported in literature. To validate our useful experience we need a randomized controlled multicentric study.
